# Cell apoptosis in ischemic stroke: Focus on lipid metabolism

**DOI:** 10.4103/NRR.NRR-D-24-00301

**Published:** 2025-01-13

**Authors:** Rong Sun, Wenren Yang, Yuting Zhao, Fumei Zhang, Genping Wu, Aiping Wang, Ying Tian

**Affiliations:** 1 Institute of Clinical Research, Department of Clinical Laboratory, Affiliated Nanhua Hospital, Hengyang Medical School, University of South China, Hengyang, Hunan Province, China; 2 Department of Biochemistry and Molecular Biology, Hengyang Medical School, University of South China, Hengyang, Hunan Province, China; 3 Department of Trauma Center, Affiliated Nanhua Hospital, Hengyang Medical School, University of South China, Hengyang, Hunan Province, China; 4 Department of Physiology, Institute of Neuroscience Research, Hengyang Medical School, University of South China, Hengyang, Hunan Province, China

**Keywords:** docosahexaenoic acid, eicosapentaenoic acid, high-density lipoprotein, low-density lipoprotein, reactive oxygen species, short-chain fatty acids, triglycerides, tumor necrosis factor

## Abstract

Ischemic stroke is a severe neurological disease with high global mortality and disability rates. Atherosclerosis has been identified as the primary cause of ischemic stroke, while abnormal lipid levels are significant contributors to the development of this condition. Multiple pro-apoptotic mechanisms are involved in ischemic stroke caused by lipid metabolism disorders, while various lipids have a strong causal relationship with neuronal apoptosis. However, studies to date have focused on the individual roles of lipid metabolism and apoptosis in ischemic stroke, and an overview of how impaired lipid metabolism leads to apoptosis in ischemic stroke is still lacking in the literature. In this review, we summarize current research on lipids in ischemic stroke. We discuss the role of lipid metabolism in accelerating apoptosis in ischemic stroke as well as the associated mechanisms. Additionally, we highlight advances in drug development and the treatment of stroke, focusing on lipid metabolism. The purpose is to provide novel ideas and strategies for the treatment and prevention of ischemic stroke.

## Introduction

**Figure 1:**
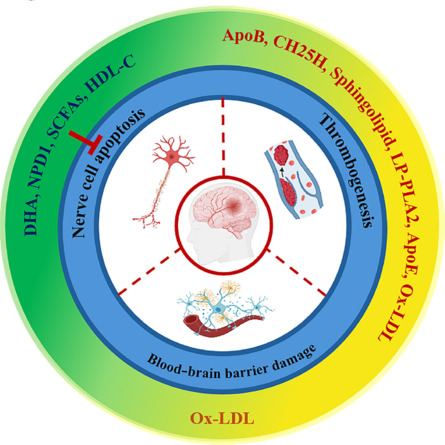
Lipids regulate the process of ischemic stroke through different pathways. DHA, NPD1, SCFAs, and HDL-C inhibit nerve cell apoptosis and protect against ischemic stroke–related damage. ApoB, CH25H, sphingolipids, LP-PLA2, ApoE, and Ox-LDL promote thrombogenesis, thereby accelerating ischemic stroke progress. Ox-LDL can also disrupt the blood-brain barrier, giving rise to ischemic stroke. Created with BioRender.com and Adobe Photoshop. ApoB: Apolipoprotein B; ApoE: apolipoprotein E; CH25H: cholesterol-25-hydroxylase; DHA: docosahexaenoic acid; HDL-C: high-density lipoprotein cholesterol; Lp-PLA2: lipoprotein-associated phospholipase A2; NPD1: neuroprotectin D1; Ox-LDL: oxidized low-density lipoprotein; SCFAs: short chain fatty acids.

**Figure 2:**
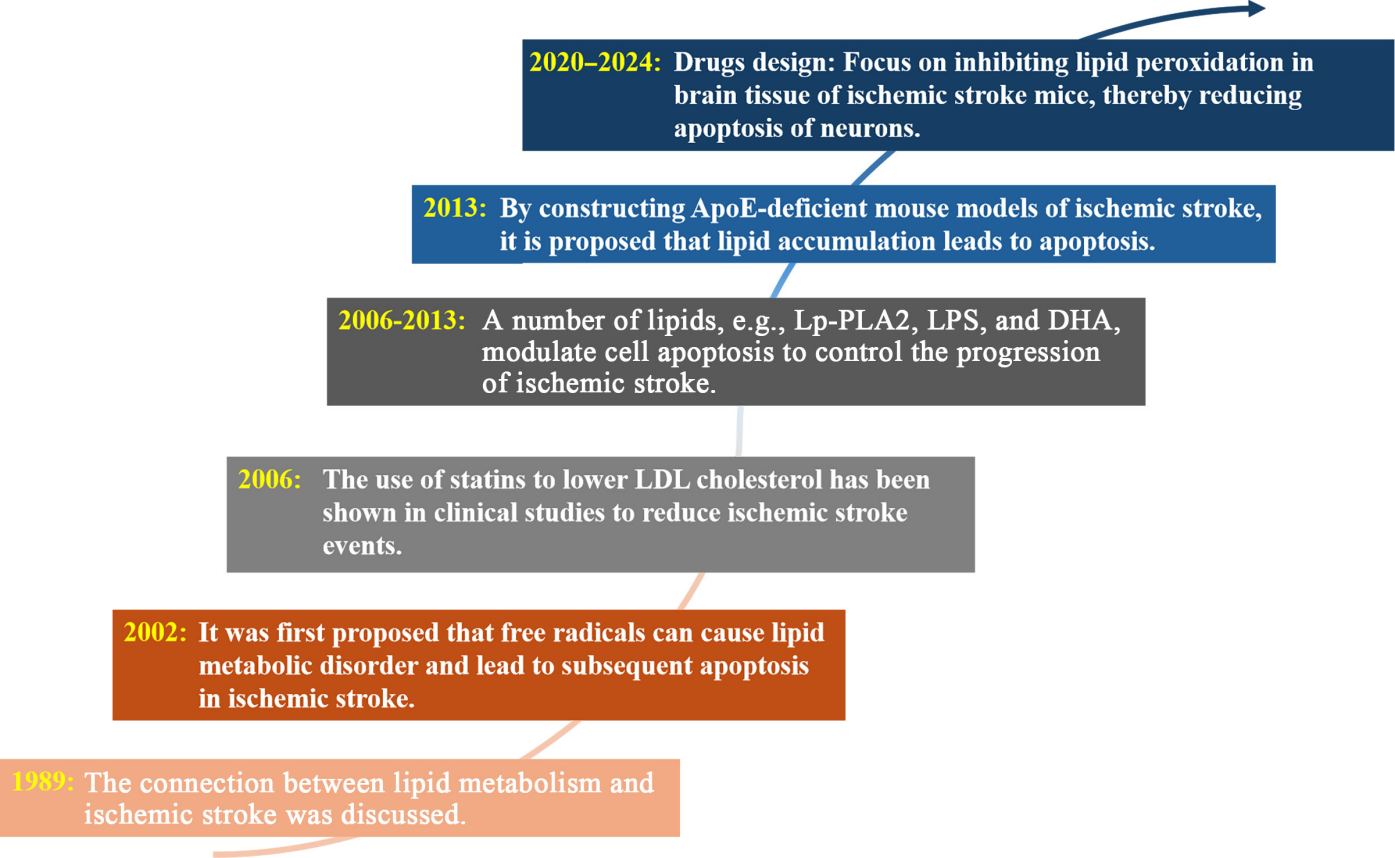
Research timeline relating to cell apoptosis and lipid metabolism in ischemic stroke. Created with BioRender.com and Adobe Photoshop. ApoE: Apolipoprotein E; DHA: docosahexaenoic acid; LDL: low-density lipoprotein; Lp-PLA2: lipoprotein-associated phospholipase A2; LPS: lipopolysaccharide.

Stroke is a severe cerebrovascular disorder associated with high morbidity, mortality, and disability rates. Stroke can be broadly divided into ischemic and hemorrhagic, with the former accounting for more than 80% of all cases (Amarenco et al., 2009). Stroke is responsible for 5.2% of all deaths worldwide and is a leading cause of disability and cognitive impairment (Hankey, 2017). However, current treatment approaches for ischemic stroke are unsatisfactory, while strategies for effectively improving neurological function are currently lacking. Thus, exploring new mechanisms and directions for the prevention and treatment of ischemic stroke is of practical importance. Ischemic stroke leads to cerebral infarction, brain tissue necrosis, focal neuronal damage, and neuronal apoptosis (Zhao et al., 2022; Wang et al., 2024; Zhang et al., 2024b). Over recent years, dyslipidemia management has become increasingly important in the treatment of this cardiovascular event, with several studies having shown that lipid metabolism disorder plays a key role in these pathological changes. For example, elevated cholesterol levels promote apoptosis by inducing an increase in the expression of cleaved caspase-3 and Bcl-2-associated X protein (BAX) and a decrease in that of B-cell lymphoma-extra large (Bcl-xL) (Li et al., 2020a). Moreover, neuronal apoptosis was reported to be significantly increased in apolipoprotein E (ApoE) knockout and dyslipidemic mice compared with that in control animals (Nguyen et al., 2022). Lipid metabolism disorders include hypercholesterolemia, decreased high-density lipoprotein (HDL) levels, increased low-density lipoprotein (LDL) levels, and hypertriglyceridemia (Rosário and Fonseca, 2023). Recent advances in human genetics, along with the results of numerous epidemiological, preclinical, and clinical trials, strongly support the existence of a causal relationship between the increase in neuronal cell apoptosis triggered by triglycerides (TG), TG-rich lipoproteins (TRL), and TRL remnants (Nagao et al., 2024) and adverse outcomes in ischemic stroke (Chapman et al., 2011). For decades, dyslipidemia has been considered a definite risk factor for atherosclerosis-induced ischemic stroke (Borén et al., 2022). Dyslipidemia-associated mechanisms are frequently linked to the apoptosis of neuronal cells. Cholesterol and its derivatives accelerate the progression of ischemic stroke by activating the pro-inflammatory response of NOD-, LRR- and pyrin domain-containing protein 3 (NLRP3) and promoting macrophage apoptosis (Zhang et al., 2024a). In addition, docosahexaenoic acid (DHA) and its metabolite, neuroprotective protein D1 (NPD1), have preventive and therapeutic effects on ischemic stroke. DHA and NPD1 inhibit the inflammatory response, Toll-like receptor (TLR) and tumor necrosis factor-alpha (TNF-α) pro-inflammatory signaling pathways, and macrophage apoptosis by suppressing the excessive production of reactive oxygen species (ROS), thereby protecting against neuronal damage in ischemic stroke (Chamorro et al., 2016). Meanwhile, deleterious increases in lipid levels lead to impaired mitochondrial function and increased peroxisomal and cytochrome oxidation by inducing oxidative stress and inflammatory responses (Sokolova et al., 2017) and directly activating pro-apoptotic pathways (Canfrán-Duque et al., 2023). These mechanisms induce the apoptosis of stroke-affected cells, such as endothelial cells, macrophages, and neurons, and promote the progression of ischemic stroke. Strategies to attenuate apoptosis in ischemic stroke by modulating dyslipidemia are particularly important. Several studies have shown that genes such as protein convertase chymotrypsin/kexin type 9 (PCSK9), APOE, and ABCA1 play significant roles in mediating the effects of lipids on ischemic stroke-related apoptosis and stroke onset (Chow et al., 2020; Li et al., 2020b; Khalil et al., 2021). The inhibition of PCSK9 reduces macrophage apoptosis by lowering plasma LDL cholesterol (LDL-C) (Prust et al., 2024), thereby effectively decreasing the risk of ischemic stroke (GBD 2021 Diseases and Injuries Collaborators, 2024). The upregulation of ABCA1 and ApoE levels via the intracerebral injection of LRX agonists can also suppress neuronal apoptosis and promote neuronal recovery after ischemic stroke (Li et al., 2014; Wang et al., 2018b). Although several recent studies have actively explored the relationship between dyslipidemia and apoptosis in ischemic stroke and have proposed new strategies for the prevention and treatment of this condition through the regulation of lipid levels (Chen et al., 2019; Amarenco et al., 2020; Banach et al., 2022; **Figures 1** and **2**), there is still a lack of relevant studies. Apoptosis resulting from lipid metabolism disorders is a significant contributor to the pathophysiology of ischemic stroke (Tang et al., 2024; Zheng et al., 2024). Importantly, ischemic stroke can be mitigated by inhibiting lipid metabolism and reducing apoptosis. In this review, we discuss the effects and mechanisms by which lipid metabolic abnormalities accelerate neuronal apoptosis and promote ischemic stroke. Additionally, we identify potential therapeutic targets and strategies for this condition involving blood lipid regulation and the protection of nerve cells.

## Retrieval Strategy

A computer-based online search of the PubMed database was undertaken to retrieve articles published up to September 31, 2024. A combination of the following text words (MeSH terms) was used to maximize specificity and sensitivity: “ischemic stroke,” “apoptosis,” “lipid metabolism,” “LDL,” “cholesterol,” “macrophage,” “neuroglia,” “atherosclerosis,” and “thrombosis.” The identified studies were further screened by title and abstract and only those exploring the relationship between lipid metabolism and cell apoptosis in ischemic stroke were included. No language or study type restrictions were applied. Articles addressing lipid metabolism in ischemic stroke without also examining apoptosis were excluded.

## Cholesterol Derivatives and Cell Apoptosis in Ischemic Stroke

Cholesterol is essential for all the cells in the human body, serving both as a major structural component of cell membranes and a substrate for the synthesis of steroids (such as bile acids and vitamin D), sex hormones (such as estradiol, progesterone, androsterone, and testosterone), and adrenocorticotropic hormones (such as aldosterone and cortisone) (Wang et al., 2017). High levels of both total cholesterol (TC) and LDL-C are associated with an increased risk of ischemic stroke, while low levels of high-density lipoprotein cholesterol (HDL-C) are a strong risk factor for this condition (Dai et al., 2021; Tang et al., 2022). Subtypes of ischemic stroke, such as atherosclerotic thrombosis and large artery occlusive disease, are strongly associated with higher TC and LDL-C levels (Jia et al., 2019; Fu et al., 2022).

### Low-density lipoprotein cholesterol and cholesterol-25-hydroxylase

**Figure 3:**
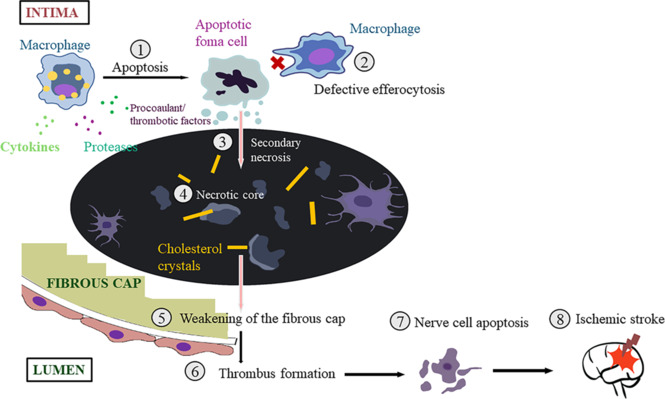
Mechanisms underlying how macrophage apoptosis leads to ischemic stroke. (1) Macrophage foam cells undergo apoptosis in response to lipid stimulation. Macrophages in advanced plaques can contribute to the formation of vulnerable plaques by secreting cytokines, proteases, and procoagulant/thrombotic factors. (2) In advanced lesions, macrophages cannot effectively clear these apoptotic cells (defective efferocytosis). This results in secondary necrosis (3) and, consequently, the formation of a necrotic core (4). (5) Smooth muscle cell death and degradation by extracellular matrix proteases can lead to the rupture of the fibrous cap. (6) Exposure to substances that cause blood clots at the lesion site can lead to thrombosis. (7) Thrombosis leads to nerve cell apoptosis. (8) Neuronal apoptosis leads to ischemic stroke. Created with BioRender.com and Adobe Photoshop.

Elevated plasma TC and LDL-C levels are important risk factors for cardiovascular and cerebrovascular diseases, including ischemic stroke, in both humans and animal models (Deng et al., 2023). High plasma cholesterol levels facilitate the infiltration and retention of lipoprotein-containing ApoB in the arterial wall. This stimulates the NLRP3 inflammasome-mediated activation of caspase-1, leading to the cleavage of pro-inflammatory cytokines of the interleukin (IL)-1 family, generating an inflammatory response, thereby increasing risk of stroke (Nègre-Salvayre and Salvayre, 2024). Moreover, increased macrophage apoptosis and the defective clearance of apoptotic cells by macrophages (efferocytosis) ultimately lead to the progression of ischemic stroke (**Figure 3**). These processes result in the deposition of residual cholesterol as plaques and the formation of cholesterol monohydrate crystals (Jin et al., 2018; Baumer et al., 2020). Cholesterol monohydrate crystals can also increase the risk of ischemic stroke by inducing macrophage apoptosis, which leads to plaque instability and rupture (Visscher et al., 2022). These studies suggest that plasma free cholesterol augments the risk of ischemic stroke by activating the inflammatory response, leading to an increase in macrophage apoptosis, and also directly induces macrophage apoptosis through the formation of cholesterol monohydrate crystals. Moreover, cholesterol derivatives increase the risk of ischemic stroke by promoting macrophage apoptosis via inflammatory responses. Other sterol intermediates and derivatives of cholesterol biosynthesis, such as oxysterols (Li et al., 2024) and 25-hydroxycholesterol (25-HC) (Adams et al., 2004; Zhang et al., 2021b), also accumulate within plaques and potentially affect the course of ischemic stroke. Cholesterol-25-hydroxylase (CH25H) generates 25-HC via cholesterol oxidation (Lund et al., 1998). Animal experiments have demonstrated that in Ch25h-knockout mice, the transformation of lipid-loaded macrophages into foam cells is delayed, and macrophages are more resistant to inflammation-induced apoptosis (Canfrán-Duque et al., 2023). The production of 25-HC by activated macrophages via CH25H amplifies their inflammatory phenotype, accelerates macrophage apoptosis, and promotes atherosclerosis, thereby increasing the risk of ischemic stroke (Canfrán-Duque et al., 2023). This experimental evidence suggests that cholesterol and its derivatives increase the risk of ischemic stroke by promoting macrophage apoptosis either directly or indirectly, the latter via inducing a pro-inflammatory response, which strongly supports the cholesterol hypothesis of apoptosis in ischemic stroke.

### Low-density lipoprotein cholesterol and high-density lipoprotein

It has long been known that cholesterol increases the risk of atherosclerosis in vascular beds, including the peripheral and coronary arteries, and that this heightens the risk of ischemic stroke. Elevated cholesterol levels can cause an ischemic stroke by facilitating arteriosclerosis development or plaque rupture. Because HDL has antioxidant and cholesterol-effluxing properties, it can promote efferocytosis resulting from ER stress (Song et al., 2016; Tekavec et al., 2020). Moreover, HDL promotes the conversion of pro-inflammatory M1 macrophages into anti-inflammatory M2 macrophages, which have a higher capacity for efferocytosis and thereby increase plaque stability (Feig et al., 2011). Following plaque rupture, HDL may also play a critical role in preventing thrombosis and platelet activation. In addition to its plaque-stabilizing effect, however, recent studies have also linked HDL to uncontrolled inflammation and the formation of susceptible plaques. Research on atherosclerotic lesions in humans has demonstrated that unstable plaques have lower levels of lipid-derived SPMs, such as lipoxin A4 (LXA4) and resolvin D1 (Fredman et al., 2016; Sun et al., 2023). These observations suggest that HDL can increase plaque stability, inhibit macrophage apoptosis, and protect against ischemic stroke through anti-inflammatory and antioxidant effects.

HDL is involved in cell survival, cell proliferation, and vasodilation (Nofer, 2015) via the triggering of cell signaling cascades. Although further research is required to determine which cell surface receptors and proteins bind to HDL, it has been shown that HDL activates many signaling cascades via multiple receptors, and its capacity for binding to the plasma membrane is well-documented. Furthermore, via cell signaling, HDL can induce the mobilization of cholesterol from intracellular stores to the plasma membrane for excretion, primarily through the HDL-mediated activation of protein kinase C (PKC) (Mendez et al., 1991; He et al., 2024b). PKC is activated by phosphatidylcholine lipase, which is activated by the binding of apoA-I to ABCA1. Activated PKC phosphorylates ABCA1, which boosts its stability and efflux activity, and increases cholesterol efflux from intracellular storage to the plasma membrane (Yamauchi et al., 2004; Kheirollah et al., 2014; Rogers et al., 2022). This exemplifies how HDL-mediated cell signaling enhances HDL cholesterol export. Notably, cholesterol absorbance by HDL also lowers the cholesterol burden in macrophages, inhibits macrophage transformation to foam cells, and suppresses atherosclerosis progression, all of which contribute to the prevention of ischemic stroke. Apart from these direct routes, HDL can also indirectly trigger cell signaling through Toll-like receptors (TLRs) (Kuwabara et al., 2014; Hata et al., 2020) or ATP (β-ATPase/P2Y12/13) (Martinez et al., 2003). Other HDL-induced signaling pathways leading to increased cholesterol and lipid efflux include the protein kinase A (PKA) (Ma et al., 2011; Preta, 2023), cell division control protein 42 (Cdc42) (Nofer et al., 2006), and Janus kinase-2 (JAK2) (Segrest et al., 2022) cascades. HDL-induced cell signaling via ABCA1 also inhibits macrophage M1 phenotype activation and proinflammatory cytokine production and promotes the secretion of M2-phenotype anti-inflammatory cytokines (e.g., IL-10) through JAK2 signaling and the activation of signal transducer and activator of transcription 3 (STAT3) (Wang et al., 2023).

Pathways downstream of HDL also have anti-ischemic stroke effects. Apolipoproteins A-I (APOA1) and A-II (APOA2) on HDLs, are determinants of HDL function with 15 and 9 proteoforms (chemical-structure variants), respectively (Lloyd-Jones et al., 2023). The ApoA-I/ABCA1/JAK2 axis enhances prostacyclin I-2 (PGI-2) production by activating cyclooxygenase-2, thus suppressing inflammation in endothelial cells (Tang et al., 2009). PGI-2 also reduces the occurrence of ischemic stroke by inhibiting atherosclerosis. Scavenger receptor class B type 1 (SR-B1) is a multiligand membrane receptor protein that functions as a physiologically relevant HDL receptor. Its main role is to mediate the selective uptake or influx of HDL-derived cholesteryl esters into cells and tissues. SR-B1 promotes the efflux of cholesterol from macrophages and peripheral tissues back to the liver. It has recently been shown that SR-BI promotes macrophage-mediated phagocytosis of dead cells and reduces neuronal apoptosis in ischemic stroke lesions through the Src/AKT/Rac1 signaling pathway (Tao et al., 2015). HDL can also induce cell signaling via SR-BI. The binding of HDL to the extracellular loop of SR-BI activates the cytosolic C-terminal domain of the latter, leading to the phosphorylation of the protein kinase Src and the activation of liver kinase B1 and calmodulin-dependent protein kinase (CAMK) (Mineo et al., 2003; Kimura et al., 2010). This results in cell signaling through downstream kinases, namely, AMP-activated protein kinase (AMPK) (Kimura et al., 2010), protein kinase B (AKT) (Mineo et al., 2003), and mitogen-activated protein kinase (MAPK) (Mineo et al., 2003), ultimately modulating angiogenesis (ubiquitin ligase Siah [Siah1/2] and hypoxia-inducible factor 1α [HIF1α]) (Li et al., 2019), revascularization (Seetharam et al., 2006), vasodilation (Zhang et al., 2012), and endothelial nitric oxide synthase (eNOS) (Ho et al., 2023). All these downstream effects contribute to some extent to HDL function, while also counteracting neuronal apoptosis in ischemic stroke. In conclusion, anti-apoptotic signaling induced by HDL and HDL-C in vascular and inflammatory cells underlies their anti-atherosclerotic and anti-ischemic stroke properties. Accordingly, HDL signaling defects may link lipid metabolism disorders with neuronal apoptosis, contributing to an increased risk of ischemic stroke.

## Fatty Acids and Cell Apoptosis in Ischemic Stroke

### Short-chain fatty acids

The mechanism underlying the pathogenicity of changes in the intestinal microbiota in ischemic stroke has attracted attention because of the proposed gut–brain axis theory (Bauer et al., 2016). Patients with both stroke and alterations in the gut microbiota have a greater number of non-neurological complications, larger infarct sizes, and worse clinical outcomes (Clottes et al., 2023). Short-chain fatty acids (SCFAs) exert protective physiological effects against ischemic stroke through the regulation of glucose metabolism (Gao et al., 2009; Tolhurst et al., 2012), inflammation (Furusawa et al., 2013), and blood pressure (Marques et al., 2018). They may also influence blood–brain barrier (BBB) stability (Ritter et al., 2024) and microglial physiology (Erny et al., 2015). SCFAs, such as butyrate, acetate, and propionate, are major metabolites in the colon, exerting immunomodulatory and neuroprotective effects, and are also an important source of energy (Stilling et al., 2016; Dalile et al., 2019). Additionally, SCFAs increase the abundance of intestinal probiotics by inhibiting TNF-α and free radicals through TLRs in the gut epithelium (Zhao et al., 2023); they also lower TMAO levels, increase the production of brain-derived neurotrophic factor (BDNF), prevent cell death, and protect against ischemic stroke (Hetman et al., 1999; Han and Holtzman, 2000). Chidambaram et al. (2022) demonstrated that SCFAs have a therapeutic effect against ischemic stroke. Furthermore, both rodent and human clinical studies have shown that a decrease in SCFA contents leads to dysregulation of the intestinal microecology, affecting various pathways associated with the development of risk factors for ischemic stroke (Pang et al., 2024). Therefore, increasing SCFA levels to boost beneficial gut bacteria, thereby achieving gut homeostasis, is critical for preventing and treating ischemic stroke and improving prognosis.

### Docosahexaenoic acid and neuroprotective protein D1

DHA is enriched in the central nervous system (Do et al., 2019) and is a precursor for the neuroprotective and homeostatic 22-carbon docosanoids and 32- or 34-carbon elovanoids (Mukherjee et al., 2004; Bazan et al., 2010). Neuronal damage subsequent to ischemic stroke can activate survival signaling pathways (Stein et al., 2024), and these can remain activated for a few hours to several days. If the ischemic brain tissue remains without reperfusion for a prolonged period, the infarct core may extend to involve the penumbra (Heiss, 2011). Subsequently, neurons in other areas of the brain may also die due to a loss of contact with ischemic neurons (secondary neuronal loss) (Kotorová et al., 2024). Therefore, the strategies for the treatment of ischemic stroke are reperfusion as soon as possible and neuroprotection, including the inhibition of neuronal apoptosis (Moskowitz et al., 2010). Aspirin is widely used to prevent cerebrovascular disease because it inhibits platelet aggregation and plays a neuroprotective role in ischemic stroke by activating DHA. In experimental stroke, DHA was shown to inhibit leukocyte infiltration and the induction of nuclear factor-kappa B (NF-κB) and cyclooxygenase-2, as well as suppress neuronal apoptosis. Additionally, DHA can lessen neuronal death by preventing IL-1β-stimulated NF-κB activation and cyclooxygenase-2 production *in vitro* (Yoshida et al., 1984). DHA also has significant inflammation-regulating activity and protects neurons from apoptosis in ischemic stroke by regulating NLRP3. The NLRP3 inflammasome is a multiprotein complex that mediates the activation of caspase-1, which results in the production of pro-inflammatory cytokines such as IL-1β and IL-18. This can lead to the apoptosis of nerve cells and accelerate the pathological process of ischemic stroke (Burris et al., 2023). DHA can decrease infarct size and brain edema by downregulating NLRP3 expression, and can also reduce neuronal apoptosis and neurological deficits, which supports the therapeutic role of DHA in ischemic stroke via the inhibition of neuronal apoptosis. Furthermore, in preclinical models of ischemic stroke, ELV, a precursor of DHA, was reported to possess neuroprotective properties and prevent neuronal death (Calandria et al., 2023). These findings demonstrate that DHA is involved in preventing brain injury and is an endogenous precursor of the mediators of the neuroprotective signaling response to ischemia–reperfusion after stroke (Calandria et al., 2023). DHA provides effective protection against neuronal damage during ischemic stroke by inhibiting inflammatory responses and reducing neuronal apoptosis. In addition, DHA can directly inhibit neuronal damage and ameliorate ischemic stroke. DHA accumulates in phospholipids that form the cell membranes of astrocytes and nerve cells on both sides of the BBB. DHA crosses the BBB into the brain parenchyma via the BBB-specific lipid transport protein major facilitator superfamily domain containing 2a (MFSD2A) and fatty acid-binding protein 5 (FABP5). In the brain, DHA accumulates in hippocampal neuronal membranes, primarily as PS-DHA. Neurons receive DHA from astrocytes, and DHA in neurons facilitates the activation of AKT and Raf-1, thereby preventing neuronal damage or apoptosis (Shen et al., 2023). The upregulation of glial cell line–derived neurotrophic factor (GDNF) can promote the proliferation and differentiation of neural stem cells in the focal cerebral cortex and hippocampus and protect against ischemic stroke-related damage (Tian et al., 2016). Numerous cohort studies have suggested that consuming large amounts of DHA may lower the risk of ischemic stroke. For example, DHA was shown to ameliorate damage caused by cerebral atherosclerosis-induced ischemic stroke through an increase in the synthesis of anti-inflammatory mediators such as resolvins (Tułowiecka et al., 2020). A previous study has also shown that DHA intake may reduce ischemic stroke mortality rates (Yamagata, 2021). Furthermore, research on older mice has demonstrated that the consumption of fish oil high in DHA and other n-3 polyunsaturated fatty acids (n-3 PUFAs) ameliorates sensory impairment in ischemic stroke via PI3K/AKT signaling and decreases neuronal apoptosis. In ischemic stroke, DHA inhibits neuronal damage through antioxidant and anti-inflammatory effects and reduces neuronal apoptosis by activating the Nrf2/HO-1 system and promoting JNK/AP-1 signaling (Rodríguez et al., 2021; Zhang et al., 2021a). Multiple studies using the mouse MCAO model have demonstrated that DHA supplementation prevents oxidative stress and inflammatory reactions linked to nerve injury. By activating the PI3K/AKT pathway, DHA also blocks neuronal death linked to cerebral infarction and brain edema (Hu et al., 2022). In summary, DHA protects against neuronal damage in ischemic stroke by directly guarding against and inhibiting neuronal apoptosis through the activation of multiple signaling pathways. The dietary intake and supply of DHA are neuroprotective against ischemic stroke, while NPD1, a metabolite of DHA, exerts a corresponding effect (Yamagata, 2021).

**Figure 4:**
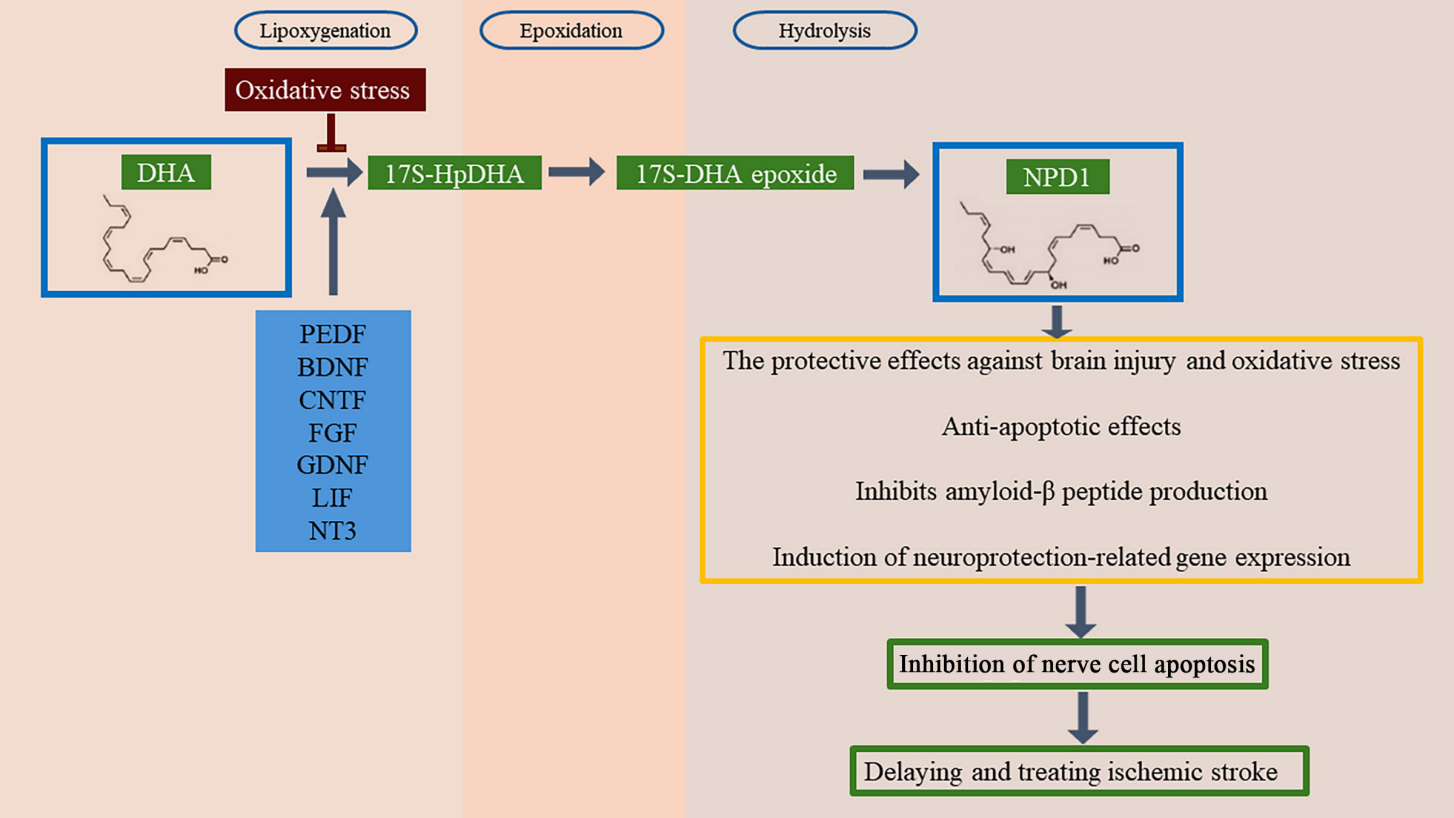
DHA can protect against ischemic stroke via the NPD1 pathway. Neurotrophins such as pigment epithelium-derived factor (PEDF), brain-derived neurotrophic factor (BDNF), ciliary neurotrophic factor (CNTF), fibroblast growth factor (FGF), glial cell–derived nerve growth factor (GDNF), leukemia inhibitory factor (LIF), and neurotrophic factor-3 (NT3) induce the biosynthesis of DHA, which is subsequently converted to NPD1. Notably, oxidative stressors inhibit NPD1 synthesis. NPD1 inhibits nerve cell apoptosis, thereby delaying and treating ischemic stroke by protecting against brain injury, oxidative stress, and apoptosis; inhibiting amyloid-β peptide production, and inducing the expression of neuroprotection-related genes. Created with BioRender.com and Adobe Photoshop. DHA: Docosahexaenoic acid; NPD1: neuroprotectin D1.

NPD1 is generated via the hydrolysis, epoxidation, and lipoxygenation of DHA. The first stage of the conversion of DHA to NPD1 is regulated by several neurotrophic factors and oxidative stressors, including pigment epithelium-derived factor (PEDF), BDNF, ciliary neurotrophic factor (CNTF), fibroblast growth factor (FGF), GDNF, leukemia inhibitory factor (LIF), and neurotrophic factor-3 (NT3). These neurotrophic factors induce the synthesis of NPD1, thereby restoring neuronal homeostasis and suppressing inflammatory and damage-inducing responses. BDNF, NGF, NT3, and other neurotrophic factors are synthetic agonists of NPD1 (Farnoodian et al., 2023). Similarly, NPD1 can downregulate cyclooxygenase-2 activity following brain nerve injury (Tu and Bazan, 2003; Serrano et al., 2011), such as ischemic stroke, as well as limit neuro-inflammatory signaling and inhibit neuronal apoptosis (Ali and Szabó, 2023). It has been demonstrated that NPD1 activity is protective against brain damage and oxidative stress in human brain neurons (Yamagata, 2021). NPD1 also reduces apoptosis in ischemic stroke by promoting the expression of the b-cell lymphoma 2 (Bcl-2) protein, thereby downregulating that of inflammatory genes and mitigating oxidative stress (Morimoto et al., 2004; Lukiw et al., 2005). Furthermore, NPD1 induces the expression of genes related to neuroprotection by promoting anti-apoptotic effects and inhibiting the production of amyloid β peptide, thereby protecting neuronal cells and exerting curative effects against ischemic stroke. NPD1 upregulates anti-apoptotic signaling in dendrites and, as a result, counteracts abnormal neuronal network activity (Bhattacharya, 2023; **Figure 4**). In conclusion, NPD1, like DHA, may enhance the treatment of ischemic stroke by inducing an anti-inflammatory response that directly or indirectly inhibits neuronal apoptosis.

### Non-esterified fatty acids

Non-esterified fatty acids (NEFAs, or free fatty acids [FFAs]) are fatty acids containing more than 10 carbons (C10). Both diabetes and obesity are non-negligible risk factors for ischemic stroke and NEFA levels are elevated in patients with obesity and type 2 diabetes (Seliga et al., 2023). NEFAs may also be involved in the development of ischemic stroke through atherosclerosis by impairing endothelium-dependent vasodilation (Steinberg et al., 1997), inhibiting endothelial nitric oxide synthase (Chen et al., 2022a), and promoting monocyte-endothelial cell adhesion through direct effects on vascular cells (Ou et al., 2022). NEFA levels are elevated in atherosclerotic disease. They promote foam cell formation by upregulating lectin-like oxidized low-density lipoprotein receptor-1 (LOX-1), leading to macrophage apoptosis, which is an important cause of ischemic stroke (Peña-Jorquera et al., 2023). Palmitic acid (PA) is the most abundant NEFA in plasma (approximately 28%) (Staiger et al., 2004). Both *in vivo* and *in vitro* studies have demonstrated that PA is involved in the pathogenesis of ischemic stroke through pro-apoptotic effects (Ishiyama et al., 2010). PA promotes the development of ischemic stroke by activating the TLR4 pathway, promoting ROS generation, inducing senescence, and triggering the apoptosis of vascular smooth muscle cells (VSMCs) and fibroblasts (Sokolova et al., 2017; Zhang et al., 2017). PA also upregulates the expression of caspase-3, caspase-9, and p53, which directly promote the apoptosis of neuronal cells, leading to atherosclerotic events, and, ultimately, ischemic stroke (Sindhu et al., 2020). These findings suggest a new strategy for treating ischemic stroke involving the inhibition of apoptotic events via a reduction in NEFA levels.

## Phospholipids and Their Oxidative Derivatives and Cell Apoptosis in Ischemic Stroke

### Sphingolipids

Phospholipids are major components of biological membranes and can be classified as glycerophospholipids or sphingolipids based on the composition of their backbone. Ceramides are a class of sphingolipids produced by the hydrolysis of sphingomyelin by sphingomyelinases, neutral sphingomyelinases, and acid sphingomyelinases (A-SMase) (Li et al., 2022). Sphingolipids and ceramides participate in the progression of ischemic stroke by accelerating atherosclerosis, increasing plaque lipid levels, and causing plaque instability (Borodzicz-Jażdżyk et al., 2022). Sphingolipids are elevated in atherosclerotic plaques and are significantly associated with the development of ischemic stroke. They are also positively correlated with inflammatory cytokines and histological markers of plaque instability. Lactose, ceramide, sphingolipids, and sphingosine 1-phosphate are associated with caspase-3 activity (Slowik et al., 1996; Schwartz et al., 2000). *In vitro* experiments revealed that glucose ceramide, lactose ceramide, and ceramide induce neuronal apoptosis and increase the risk of ischemic stroke by activating the caspase-3-mediated apoptotic pathway (Martin et al., 2006). Furthermore, VSMCs are important for maintaining plaque stability (Lee et al., 2024). The loss of stable VSMCs is a key feature of vulnerable plaques, leading to the thinning of the protective cap and significantly promoting subsequent plaque rupture. This results in the apoptosis of macrophages and the cells that constitute the main feature of unstable plaques and increases the risk of ischemic stroke. Lactose ceramide and ceramide mediate A-SMase-induced apoptosis in human osteosarcoma cell lines and endothelial cells (Kubo et al., 2007; Sarlon-Bartoli et al., 2012). However, ceramide and lactose ceramide are negatively correlated with the plaque VSMC constriction (Slowik et al., 1996; Schwartz et al., 2000). These contrasting effects on ischemic stroke may result from differences in pathological and physiological states in which ceramides and lactose ceramides delay unstable plaque formation and ischemic stroke by reducing the plaque VSMC content. Notably, under pathological conditions, several ceramides accelerate the progression of ischemic stroke by activating the caspase-3-mediated apoptotic pathway and inducing endothelial cell apoptosis, although the exact underlying mechanism remains to be elucidated. In conclusion, these studies demonstrated that sphingolipids and their derivatives regulate plaque lipid levels, influence vascular endothelial cell stability, and activate apoptotic pathways in VSMCs and neuronal cells, highlighting their potential as novel therapeutic targets in ischemic stroke.

### Lipoprotein-associated phospholipase A2

Lipoprotein-associated phospholipase A2 (Lp-PLA2), also known as platelet-activating factor acetylase, is an inflammatory cell-secreted phospholipase that can induce the hydrolysis of oxidized phospholipids. Lp-PLA2 rapidly cleaves oxidized phosphatidylcholine molecules generated during the oxidation of LDLs and lipoprotein Lp(a) and generates soluble pro-inflammatory and pro-apoptotic lipid mediators. These pro-inflammatory lipids play an important role in the formation of the atherosclerotic necrotic core and matrix in acute unstable coronary artery disease by recruiting and activating leukocytes/macrophages, inducing apoptosis, and influencing the subsequent clearance of dead cells. Notably, Lp(a) serves as an independent risk factor for cardiovascular and cerebrovascular diseases. While its levels are predominantly determined by genetic factors, with minimal influence from the environment, lipid-lowering medications, such as statins, can also modestly increase Lp(a) levels (Tsimikas et al., 2020a, b). Lp(a), an acute-phase reactant, may play a significant role in acute cardio-cerebrovascular events (Maeda et al., 1989; Nakamura et al., 2020). Lp(a) has a potential prothrombotic effect, inhibiting fibrinolysis and tissue factor pathway inhibitor (TFPI) activation by increasing PAI-1 expression levels, thus enhancing coagulation factor VII activation and promoting blood coagulation (Buechler et al., 2001). This may give rise to an unstable shedding of atherosclerotic plaques, leading to the occurrence of ischemic stroke. Given these findings, elevated levels of circulating Lp-PLA2 have emerged as a risk marker for the development of ischemic stroke (Kruth et al., 2002; Finn et al., 2012). Patients with high carotid stenosis have increased circulating Lp-PLA2 levels, which can be used as an independent indicator of plaque instability. Unstable plaque shedding is also a primary pathogenic factor in ischemic stroke (Benitez et al., 2023), implying that Lp-PLA2 may similarly serve as an independent predictor of the risk of this condition. The selective inhibition of Lp-PLA2 reduces necrotic core formation and stabilizes atherosclerotic plaques, thereby preventing ischemic stroke, while also decreasing macrophage apoptosis and delaying the progression of the necrotic core in ischemic stroke (Zheng et al., 2016). Lp-PLA2 can be used to treat the clinical sequelae of late ischemic stroke by reducing nerve cell apoptosis (Roeters van Lennep et al., 2023). Lp-PLA2 specifically hydrolyzes oxidized phospholipids in LDL, yielding two potent pro-inflammatory mediators, namely, lysophosphatidylcholine (LPC) and oxidized NEFA (Widmer et al., 2019). Lp-PLA2-generated LPC is a potent inducer of monocyte chemotaxis, promoting foam cell aggregation in the arterial wall, macrophage apoptosis, and ischemic stroke (Zhang et al., 2022a). In addition to being present in the circulation, Lp-PLA2 is also expressed in human coronary plaques. Lp-PLA2 is particularly abundant in lesional macrophages and foam cells around the necrotic core of advanced ischemic stroke lesions prone to rupture, suggestive of its importance in inducing macrophage apoptosis and accelerating ischemic stroke progression (Albert et al., 2005; Moutzouri et al., 2013). In a model of ischemic stroke caused by aortic atherosclerosis, darapladib selectively inhibited Lp-PLA2, accompanied by a significant reduction in the levels of related indicators of macrophage activation (Widmer et al., 2019). This provided evidence that Lp-PLA2 promotes macrophage apoptosis through an inflammatory response and accelerates ischemic stroke by limiting the expansion of the necrotic core in ischemic stroke plaques (Crombag et al., 2019). Additionally, brain injury induces glutamate excitotoxicity and ROS accumulation, leading, in turn, to early PLA2 activation via upstream kinases such as ERK1/2 and p38 MAPK (Lu et al., 2019). PLA2 can promote lysosomal membrane permeabilization (LMP) in cell lines and primary neurons (Lipinski et al., 2015; Jassam et al., 2017). Its initial neuron-specific activation contributes to lysosomal damage and subsequent neuronal apoptosis. However, PLA2 activation in microglia and/or infiltrating macrophages at a later time (3 days) after stroke is likely a response to inflammatory signaling (Loane and Faden, 2010). *In vivo* pharmacological inhibition of PLA2 was shown to attenuate brain injury–induced LMP and the subsequent impairment of autophagy and neuronal loss and was associated with improved neurological outcomes (Sarkar et al., 2020). In addition, many hemolytic dopamine products generated by Lp-PLA2 were observed in ischemic stroke-induced carotid plaques and were associated with increased levels of pro-inflammatory cytokines. These findings suggest that Lp-PLA2 leads to plaque inflammation, decreases plaque lesion stability, and is important in inducing unstable plaque formation and macrophage apoptosis, which may lead to ischemic stroke (Saam et al., 2013). In essence, the localized production of Lp-PLA2 by arterial macrophages and foam cells may be a more significant contributor to atherosclerosis and the induction of ischemic stroke than the levels of circulating Lp-PLA2. Overall, these findings indicate that Lp-PLA2 expression is positively correlated with the necrotic core area, macrophage number, and neuronal apoptosis in ischemic stroke.

## Low-density Lipoprotein and Its Oxidative Derivatives and Cell Apoptosis in Ischemic Stroke

LDL and HDL combine with cholesterol to form LDL-C and HDL-C, respectively, and are involved in the transport, decomposition, recycling, and excretion of cholesterol. Controlling LDL-C levels is the primary goal of lipid modification therapy in patients with stroke. Mechanistically, LDL functions much like HDL, mainly transporting cholesterol into cells. However, excess LDL leads to cholesterol accumulation in the arterial wall, resulting in arterial stiffness and related cell apoptosis, which are important causes of ischemic stroke.

### Oxidized low-density lipoprotein

Oxidized LDL (Ox-LDL) is derived from the oxidative modification of LDL. The oxidative stress theory suggests that oxidative modification of LDL is critical for initiating and accelerating atherosclerotic lesion formation, leading to the development of ischemic stroke (Ross, 1999). Increased ROS production within the vascular wall is linked to factors such as smoking, diabetes, hypertension, and dyslipidemia, which increase the risk of ischemic stroke (Su et al., 2024). LDL is converted to Ox-LDL as ROS levels increase. Ox-LDL induces ischemic stroke by acting on fibroblasts, endothelial cells, and macrophages via the transmembrane glycoprotein LOX-1 (Xu et al., 2013).

Ox-LDL activates NF-κB through LOX-1, which, in turn, triggers the inflammatory response and increases LOX-1 expression. This results in a vicious cycle of elevated ROS production, increased Ox-LDL absorption, and elevated LOX-1 receptor expression (Dandapat et al., 2007). The interaction between Ox-LDL and LOX-1 promotes monocyte adherence to endothelial cells, which increases the production of cytokines such as MCP-1 and adhesion molecules such as VCAM, and also induces endothelial cell death and MAPK activation, which are crucial pathogenic processes in ischemic stroke (Singh and Gautam, 2019). The Ox-LDL/LOX-1 pathway also leads to increased MCP-1 expression and monocyte adhesion via MAPK activation. This promotes monocyte-macrophage migration and differentiation and subsequent macrophage apoptosis, together with increased expression of cell adhesion molecules such as VCAM-1 and ICAM-1, which is a key step in atherogenesis leading to ischemic stroke. In addition, the induction of LOX-1 by Ox-LDL reduces the phosphorylation of AKT, a kinase that mediates eNOS activation through phosphorylation (Bao et al., 2014). Reduced AKT phosphorylation due to decreased eNOS activation diminishes NO production. NO deficiency can lead to vasoconstriction dysfunction as well as promote endothelial cell apoptosis, another important cause of ischemic stroke. Moreover, LOX-1 contributes to the upregulation of angiotensin-converting enzyme expression and the activation of the NLRP3 inflammasome, both of which accelerate the progression of ischemic stroke and induce apoptosis in related cells (Hofmann et al., 2023). Furthermore, Ox-LDL upregulates the intrinsic apoptotic pathway by activating caspase-3 and caspase-9, suppressing the synthesis of anti-apoptotic proteins such as a cellular inhibitor of apoptotic protein 2 (c-IAP-2) and Bcl-2, promoting neuronal apoptosis, and aggravating ischemic stroke (Zhang et al., 2022b). In Ox-LDL-treated human coronary artery endothelial cells, mitochondria were observed to release activators of caspase-9, cytochrome c, and SMAC into the cytoplasm (Salvayre et al., 2002), likely because Ox-LDL upregulates FAS expression on the endothelial cell surface, activating the exogenous apoptotic pathway and resulting in FAS-mediated apoptosis (Jackson et al., 2022).

Ox-LDL also induces the secretion of inflammatory cytokines such as TNFα, IL-1, MCP-1, and IL-8 from macrophages, which subsequently activate other types of inflammatory cells (Jovinge et al., 1996; Botham and Wheeler-Jones, 2013; Wilhelm et al., 2023). Ox-LDL can polarize macrophages into M1-like phenotypes depending on the degree of oxidation (de la Paz Sánchez-Martínez et al., 2017). In addition, Ox-LDL promotes the chemotaxis of monocytes, neutrophils, eosinophils, and T cells (Han et al., 2004) to the arterial wall. Subsequently, Ox-LDL induces macrophage apoptosis and the formation of unstable plaques that are prone to rupture (Savitskaya et al., 2022). Ischemic stroke after plaque rupture is a key cause of death. Thus, this suggests that elevated levels of circulating Ox-LDL may increase the risk of death from ischemic stroke. In conclusion, through both endogenous and exogenous mechanisms, Ox-LDL accelerates the course of ischemic stroke and promotes the apoptosis of macrophages and endothelial cells.

### Apolipoprotein E

ApoE is a major lipid and cholesterol carrier in the central nervous system and is closely related to lipoprotein metabolism. ApoE is polymorphic, and its polymorphisms are closely associated with the occurrence and development of ischemic stroke through atherosclerosis. In addition, ApoE is involved in regulating neuronal damage in ischemic stroke by participating in the activation of lipid-hydrolyzing enzymes, immune regulation, and neuronal tissue regeneration. The APOE gene has three alleles (E2, E3, and E4) that give rise to six genotypes (E2/2, E2/3, E2/4, E3/3, E3/4, and E4/4) (Moriarty et al., 2020). It has been shown that the presence of ApoE4 is associated with a higher risk of cardiovascular disease and dementia compared with the other subtypes (Chen et al., 2022b). People of African descent have a higher frequency of the APOE4 genotype than Caucasian individuals, and their mean Lp(a) levels are also two to four times higher (Howard et al., 1998). The frequency of the E4 allele may be up to twice as high in people of African descent relative to that in people of European and Asian descent (Goldstein et al., 2020). ApoE4 is also associated with higher levels of proinflammatory cytokines and the promotion of thrombosis and is less effective than ApoE3 at enabling the efferocytosis of apoptotic cells (Cash et al., 2012). ApoE4 accelerates the risk of stroke likely through its disruptive effects on the BBB (Austin, 1991; Duewell et al., 2010). A meta-analysis conducted by the Swedish Heart and Lung Foundation on the interaction between APOE alleles and sex, age at ischemic stroke onset, stroke severity, and outcome revealed a direct association between male carriers of the APOE2 allele and adverse stroke outcome (Lagging et al., 2019). Brain-like organoids derived from patients with neurological disorders who carry APOE4 exhibit increased levels of apoptosis and reduced synaptic integrity (Chen et al., 2021b).

Recent evidence suggests that ApoE influences the central nervous system response to acute and chronic injury, promotes the release of histamine and inflammatory mediators, exacerbates neuronal injury, and enhances neuronal apoptosis, thus contributing to adverse outcomes in ischemic stroke (Hoffmann et al., 2024). ApoE promotes the conversion to the inflammatory (M1) phenotype of macrophages by mediating the uptake of very-low-density lipoprotein (VLDL) and its remnants, enhancing inflammation, increasing phagocytosis and foam cell formation, and impairing efferocytotic activity (Liberale et al., 2017; Pourcet and Staels, 2018). This stimulates macrophage apoptosis, thereby accelerating the course of ischemic stroke, accompanied by the activation of metalloproteinase expression, which contributes to fibrous cap thinning, another causative factor of ischemic stroke (Laskowitz et al., 2010). Notably, ApoE is also abundantly present in the central nervous system. After ischemic stroke, ApoE exerts neuroprotective effects by mitigating excitotoxicity and inhibits neuronal apoptosis (Gorelick and Mazzone, 1999; Mace et al., 2007). This highlights the need to account for the differing effects of ApoE before and after stroke, with a focus on rationally regulating its expression to inhibit inflammatory responses, delay macrophage apoptosis, and protect against neuronal damage. Achieving this remains an important challenge in managing patients with ischemic stroke.

## Regulation of Lipid Metabolism in Ischemic Stroke

**Figure 5:**
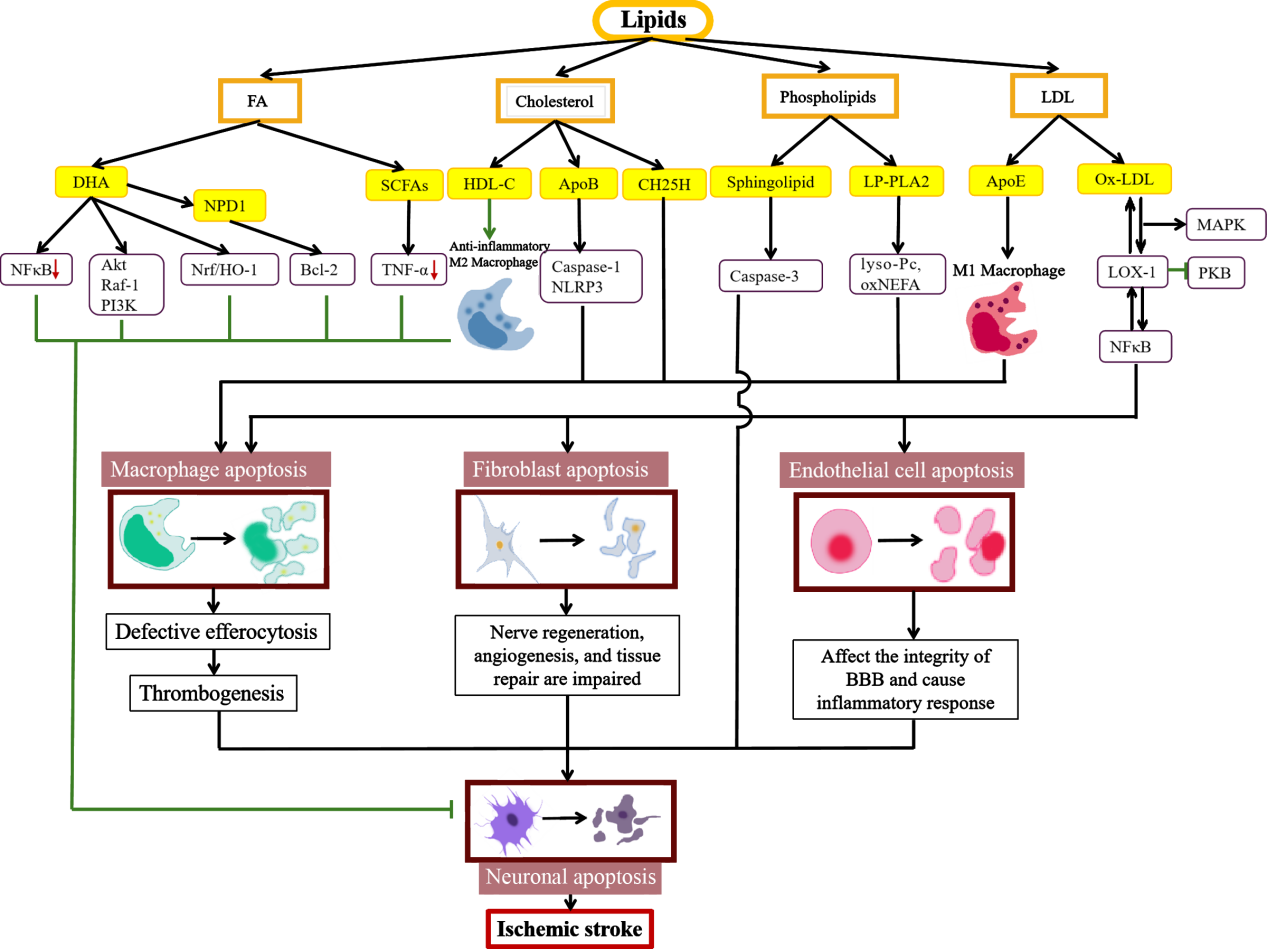
Multiple lipids promote the progression of ischemic stroke by activating pathways that accelerate nerve cell apoptosis. The lipid substances discussed in this review include FA, cholesterol, phospholipids, LDL, and their derivative. These lipids are involved in regulating cell apoptosis in ischemic stroke through diverse signaling pathways. Notably, ApoE promotes macrophage apoptosis by stimulating macrophage conversion to the M1 phenotype. ApoB: Apolipoprotein B; ApoE: apolipoprotein E; BBB: blood–brain barrier; Bcl-2: B-cell lymphoma 2; CH25H: cholesterol-25-hydroxylase; DHA: docosahexaenoic acid; FA: fatty acids; HDL-C: high-density lipoprotein cholesterol; LDL: low-density lipoprotein; LOX-1: lipoprotein receptor-1; Lp-PLA2: lipoprotein-associated phospholipase A2; MAPK: mitogen-activated protein kinase; NEFA: non-esterified fatty acids; NF-κB: nuclear factor-kappa B; NPD1: neuroprotectin D1; Ox-LDL: oxidized LDL; PKB: protein kinase B; SCFAs: short chain fatty acids; TNF-α: tumor necrosis factor α.

Dyslipidemia has been implicated in the pathogenesis of ischemic stroke. Multiple lipids promote the ischemic stroke pathway by inducing apoptosis in diverse cell types (**Figure 5** and **Table 1**). Elevated TC, TG, LDL-C, and HDL-C concentrations are considered risk factors and predictors of stroke (Jain et al., 2013; Holmes et al., 2018). Besides standard lipid composition assays, the assessment of lipid and lipoprotein composition, particle size, and density has been proposed as a means for the detection of subclinical disease (Tziomalos et al., 2009). Dyslipidemia is an important independent risk factor for stroke (Wang et al., 2017), and modulating lipid metabolism via the lowering of cholesterol levels is the primary strategy for the prevention of ischemic stroke. The mechanisms and pathways that have been demonstrated to regulate several important types of lipid metabolism, in both clinical and other studies, are described below.

**Table 1:** Summary of the multiple lipids and mechanisms associated with ischemic stroke

Lipid		Key role	Mechanism
FAs	DHA	Beneficial	1. Lessen neuronal death by preventing NF-κB activation and cyclooxygenase-2 production in vitro, which are stimulated by interleukin-1β.
			2. Decrease neuronal apoptosis by activating AKT, Raf-1, PI3K/AKT, Nrf2/HO-1, and JNK/AP-1.
			3. Convert DHA to NPD1, which upregulates Bcl-2 expression, thereby protecting against oxidative stress in ischemic stroke.
	SCFAs	Beneficial	Increase intestinal probiotic abundance by inhibiting TNF-alpha and free radicals through TLRs in the gut epithelium; increase brain-derived neurotrophic factor expression; prevent cell death; and protect against ischemic stroke.
Cholesterol	HDL-C	Beneficial	Promote the conversion of macrophages to an M2 phenotype; M2 macrophages have a higher capacity for efferocytosis and thereby increase plaque stability.
	ApoB	Detrimental	The infiltration and retention of ApoB in the arterial wall stimulates caspase-1 activation by the NLRP3 inflammasome and generates an inflammatory response in stroke.
	CH25H	Detrimental	The production of 25-HC by activated macrophages via CH25H amplifies the inflammatory phenotype of macrophages, accelerates macrophage apoptosis, and promotes atherosclerosis.
Phospholipids	Sphingolipid	Detrimental	Activate the caspase-3-related apoptotic pathway.
	Lp-PLA2	Detrimental	Lp-PLA2-generated LPC leads to foam cell aggregation in the arterial wall and accelerates macrophage apoptosis in ischemic stroke.
LDL	ApoE	Detrimental	Activate LOX-1, increase NLRP3 and ROS contents, induce the secretion of inflammatory cytokines, and polarize macrophages to an M1 phenotype.
	Ox-LDL	Detrimental	1. Ox-LDL activates NF-κB through LOX-1, which, in turn, activates the inflammatory response and increases LOX-1 expression.
			2. Interaction between Ox-LDL and LOX-1 leads to the activation of endothelial cell death and MAPK, both of which are crucial pathogenic processes in ischemic stroke.

Bcl-2: B-cell lymphoma 2; CH25H: cholesterol-25-hydroxylase; DHA: docosahexaenoic acid; FA: fatty acids; HDL-C: high-density lipoprotein cholesterol; LOX-1: lectin-like oxidized low-density lipoprotein receptor-1; LPC: lysophosphatidylcholine; Lp-PLA2: lipoprotein-associated phospholipase A2; MAPK: mitogen-activated protein kinase; NLRP3: NOD-, LRR- and pyrin domain-containing protein 3; NPD1: neuroprotectin D1; PI3K: phosphatidylinositol 3-kinase; ROS: reactive oxygen species; SCFAs: short chain fatty acids; TLR: Toll-like receptor; TNF-alpha: tumor necrosis factor-alpha.

### Cholesterol regulation

Substantial evidence supports that elevated total and LDL cholesterol levels in plasma are an important risk factor for the development of cerebrovascular disease in both humans and model animals (Wang et al., 2017). Reverse cholesterol transport involves the HDL-mediated removal of excess cholesterol that has accumulated in peripheral tissues and its transportation to the liver for excretion into the feces via bile (Luo et al., 2022). Plasma HDL is the smallest lipoprotein particle and displays a higher apolipoprotein-to-lipid ratio relative to LDL, VLDL, and chylomicrons (Zhang et al., 2014). The major HDL-associated proteins, apoA-I and apoA-II, are secreted into plasma by the liver and the intestine, and are subsequently lipidated, forming lipid-poor, discoidal, nascent HDL (Wang et al., 2017), which takes up cholesterol from cell membranes and other lipoproteins. This implies that the regulation of cholesterol levels may be an effective strategy for the treatment of ischemic stroke. Clinical trials for novel lipid-modifying agents, including inclisiran, bempedoic acid, and pemafibrate, are currently underway (Hackam and Hegele, 2022). Cholesterol-lowering drugs, such as statins, inhibit cholesterol biosynthesis in the liver by suppressing the enzymatic activity of HMG-CoA reductase (Tsivgoulis et al., 2015). They also increase the density of LDL receptors in the liver, which helps clear LDL from the blood and reduces the risk of ischemic stroke (Gold et al., 2012). A compelling meta-analysis showed that the further lowering of LDL-C levels safely reduces the rates of heart attack, revascularization, and ischemic stroke (Baigent et al., 2010). Each 1 mM decrease in LDL-C concentrations was shown to decrease the annual rate of these major vascular events by just over a fifth. A reduction of 2 to 3 mM in LDL-C would diminish risk by between 40% and 50%. Statin therapy has been shown to reduce the rate of major cardiovascular and cerebrovascular events in a wide range of individuals. Standard statin therapy or more intensive statin regimens were associated with a 25% reduction in the risk for coronary revascularization procedures for every 1 mM decrease in LDL-C. However, the reductions in the risks of major coronary events were less pronounced among older adults. The proportions of reductions in cardiovascular and cerebrovascular events associated with statin use were lower among older patients than among younger patients without known vascular disease. The proportion of cardiovascular deaths was reduced by 12% for each 1 mM decrease in LDL-C. The protective effect of LDL-C reduction on cardiovascular disease-related mortality decreased with increasing age (Cholesterol Treatment Trialists’ Collaboration, 2019). Clinically, atorvastatin is effective in patients with previous transient ischemic attack and non-cardioembolic ischemic stroke. Pretreatment with statins is associated with better clinical outcomes in patients with ischemic stroke (Fuentes et al., 2009). Statins and ezetimibe reduce the risk of ischemic stroke without increasing that of hemorrhagic stroke. PCSK9 inhibitors similarly reduce ischemic stroke risk in statin-treated patients with atherosclerosis without increasing hemorrhagic stroke, even when very low levels of LDL-C are achieved (Di Costanzo et al., 2023). The inhibition of PCSK9 is a therapeutic strategy for reducing plasma LDL-C levels. *In vivo* experiments demonstrated that PCSK9 overexpression promoted the accumulation of LDL-C in plasma of mice, whereas PCSK9 knockout resulted in hypocholesterolemia (Rashid et al., 2005). The mechanism involves the endocytosis of PCSK9 through its binding to LDLR on the cell surface. Combined LDLR is unable to change the conformation of the inner nuclear body. Subsequently, LDLR and LDL particles are transported to lysosomes for degradation (Hazen, 2012). PCSK9 may also lower TG levels by modulating the expression of free fatty acid transporter proteins (Kloska et al., 2020). Research has demonstrated that melatonin can effectively reduce pro-inflammatory reactions and lipid peroxidation (Maity et al., 2023), thereby preserving the integrity of blood vessels and lessening free radical-induced apoptosis (García-García et al., 2009). By preventing cholesterol production and encouraging cholesterol catabolism, long-term melatonin therapy keeps blood cholesterol levels and body weight in check. Furthermore, melatonin promotes the polarization of macrophages into an anti-inflammatory state, acting as a suitable barrier during the recovery phase of ischemic stroke (Bouhlel et al., 2007; Cochain and Zernecke, 2015). Moreover, we and others have shown that the overexpression of IDOL in cells can hinder the uptake of LDL by reducing the protein levels of LDLR, resulting in hypercholesterolemia (Choi et al., 2020), and that the overexpression of IDOL can inhibit LDL uptake by downregulating the protein level of LDLR, leading to hypercholesterolemia (Sasaki et al., 2014). A previous study employing human liver and mouse macrophage cell lines has reported that plasma FGF21 can reduce the mRNA and protein levels of IDOL and the blood cholesterol content (Do et al., 2012). Thus, the application of statins, increased ATF3 expression, melatonin supplementation, and the knockdown of PCSK9 and IDOL can effectively reduce cholesterol levels and alleviate abnormalities in lipid metabolism (Liu and Vaziri, 2014; Chen et al., 2017; Guo et al., 2023).

Studies have shown that the inhibition of acyl-coenzyme A: cholesterol transferase (ACAT) can limit the absorption of cholesterol and reduce the secretion of apolipoprotein B-containing lipoproteins, such as VLDL, thereby reducing plasma cholesterol levels (Leon et al., 2005; Chang et al., 2006; Tian et al., 2022). This underscores the potential of ACAT as a novel therapeutic target for ischemic stroke. Elevated levels of FFAs in the blood lead to enhanced TG synthesis in the liver and, consequently, an increase in VLDL levels (Wierzbicki, 2004). ACAT is an intracellular enzyme that catalyzes the esterification of cholesterol and FFAs to cholesteryl esters. The accumulation of cholesteryl esters is a key process in the transformation of macrophages into foam cells and is closely related to the formation of atherosclerotic plaques. Inhibiting ACAT reduces the accumulation and deposition of cholesteryl esters and increases the clearance of free cholesterol in macrophages and vessel walls (Aflaki et al., 2011). Macrophages undergo apoptosis when safety mechanisms that prevent the accumulation of excess free cholesterol fail. This indicates that ACAT accelerates the progression of ischemic stroke by promoting the synthesis of cholesteryl esters and the apoptosis of macrophages. Moreover, in macrophages, energy deprivation due to defective lipolysis can also result in the spontaneous induction of apoptosis (Yao and Tabas, 2001). FFAs ingested by macrophages must first be esterified to TG via the hydrolytic activity of adipose triglyceride lipase (ATGL) before they can be used as an energy substrate (Chandak et al., 2010). *In vitro* experiments have shown that macrophages lacking ATGL have reduced ATP, total acyl-coenzyme, and acylcarnitine concentrations, while TG accumulation triggers neuronal apoptosis (Chang et al., 2006), a hallmark of pathological changes in ischemic stroke. Combined, the above studies have confirmed that inhibiting ACAT activity can serve as a therapeutic strategy for ischemic stroke by reducing serum cholesterol levels as well as macrophage and nerve cell apoptosis.

### Regulation of n-3 polyunsaturated fatty acids

The regulation of n-3 PUFAs is essential for maintaining normal biological activity and function in living organisms. These fatty acids are challenging to synthesize in the human body and must be supplemented orally (Ueno et al., 2019). Fish are the main dietary source of n-3 PUFAs in the form of DHA, docosapentaenoic acid, and eicosapentaenoic acid (EPA) (Thies et al., 2003). Studies indicate that patients taking fish oil experienced plaque regression, accompanied by an increase in the contents of EPA and DHA within the plaque and a decrease in the number of macrophages (Ajami et al., 2011). DHA and EPA levels can be supplemented by the consumption of fish oil (Bazan, 2007, 2009; Yamagata, 2021). This increases the levels of the anti-apoptotic proteins Bcl-2 and Bcl-xL, which inhibit the inflammatory response and protect against ischemic brain injury (Mozaffarian and Wu, 2011). The strong anti-inflammatory characteristics of n-3 PUFAs serve to prevent platelet aggregation, stabilize atherosclerotic plaques, and reduce the risk of ischemic stroke due to several risk factors, including hypertension and hyperlipidemia (Rebiger et al., 2016). As antioxidants, n-3 PUFAs can reduce brain lipid peroxides and modulate oxidative stress caused by lipid disorders in ischemic stroke (Serhan, 2017). Following stroke, n-3 PUFAs can also elicit other responses, such as neurogenesis and hemodialysis, and may be used in the development of ischemic stroke therapies (Thies et al., 2003). Additionally, aspirin is commonly used in treating ischemic stroke because it inhibits platelet aggregation, promotes thrombolytic recanalization, and exerts neuroprotective effects in ischemic stroke by activating DHA (Berger et al., 2005). Therefore, increasing n-3 PUFA content via supplementation with fish oil or drug administration can effectively alleviate the oxidative stress caused by dyslipidemia and regulate dyslipidemia in ischemic stroke.

### Regulation of free fatty acids

Numerous studies have analyzed the impact of FFAs on the pathogenesis and risk of stroke and have highlighted the important role played by FFAs in the pathogenesis of cognitive dysfunction in patients with ischemic stroke (Barberger-Gateau et al., 2007; Kotlęga et al., 2021). FFA contents in cerebrospinal fluid can serve as an independent biomarker for evaluating the prognosis and clinical outcome of ischemic stroke (Gaber et al., 2020). Peroxisome proliferator-activated receptor (PPAR) is crucial in maintaining FFA and glucose homeostasis, making it a promising target for dyslipidemia treatment (Lewis et al., 2002; Evans et al., 2004). Rosiglitazone, a PPARγ agonist, is commonly used to treat type 2 diabetes (Staels and Fruchart, 2005). However, clinical data suggest that both rosiglitazone and pioglitazone, another PPARγ agonist, can affect blood glucose levels and lipid parameters (Hauner, 2002; Khan et al., 2024). Multiple studies have shown that the use of rosiglitazone can significantly improve neurological function and reduce the brain infarct volume by 72 hours after stroke (Chen et al., 2021a). PPARγ is primarily expressed in adipose tissue. Its activation enhances glucose uptake and storage, as well as the absorption of FFAs in adipose tissue (He et al., 2024a). This leads to a reduction in blood FFA levels, the lowering of VLDL levels, and the alleviation of dyslipidemia. Thus, PPARγ can effectively alleviate dyslipidemia-induced atherosclerosis and prevent ischemic stroke.

### Regulation of apolipoprotein E

Current therapeutic strategies for reducing ApoE4 levels include directly targeting ApoE4 or converting it to other isoforms whenever possible, ApoE4 lipidation, anti-ApoE4 immunotherapy, antisense oligonucleotide therapy, and other non-pharmacological approaches, such as dietary changes and physical activity (Fernández-Calle et al., 2022). Applying technologies such as single-cell sequencing and novel methods such as synaptometry by time of flight, along with the use of female and male animal models of ApoE and consideration of ApoE genotypes, will be valuable for optimizing the application of lipid-lowering therapies that modulate ApoE in ischemic stroke (Williams et al., 2023). Experimental and clinical studies on the effective modulation of ApoE expression in the brain to regulate dyslipidemia in ischemic stroke are currently lacking.

### Regulation of high-density lipoprotein

Soluble sphingolipid sphingosine-1-phosphate (S1P), a key physiologically active lipid on HDL, mediates most HDL signaling activation. The majority of S1P in the bloodstream is linked to HDL, and HDL-S1P activates G protein-coupled S1P receptors (S1P1–5) found on the surface of several vascular cell types, such as smooth muscle cells, endothelial cells, and macrophages. Several signaling cascades and components that directly support many of the antiatherogenic qualities of HDL, including increased endothelial barrier function (Therond and Chapman, 2022) and angiogenesis (Miura et al., 2003; Therond and Chapman, 2022), along with concomitant reductions in inflammation (Miura et al., 2003) and apoptosis in ischemic stroke (Nofer et al., 2001), are triggered by activation of the S1P1 and S1P2 receptors. HDL also inhibits smooth muscle migration via S1P signaling. S1P signaling is a key factor in restenosis and plaque formation (Tamama et al., 2005). These are all important steps in the development of atherosclerosis, which eventually results in ischemic stroke. Notably, HDL-S1P levels are decreased in patients with cerebrovascular and cardiovascular illness (Sattler et al., 2015). Focal adhesion kinase, NF-κB, nicotinamide adenine dinucleotide phosphate (NADPH) oxidase, eNOS, STAT3, and Bcl-xL (Nofer, 2015) are the main end-effectors in the S1P-activated G protein-coupled receptor signaling cascades. HDL-S1P signaling has additionally been reported to exert anti-apoptotic and vasodilatory effects (Morel et al., 2012; Rusiecka et al., 2020). HDL has many beneficial properties, including anti-inflammatory, anti-oxidative, anti-thrombotic, anti-infective, anti-apoptotic, and pro-vasodilatory functions, and is also involved in intercellular communication. Drugs such as torcetrapib, evacetrapib, dalcetrapib, and anacetrapib have been evaluated in phase III clinical trials for their potential to raise HDL-C levels and regulate lipid metabolism disorders, and have shown promising results, including improving outcomes in ischemic stroke (Wang et al., 2018a).

## Limitations

This review has some limitations, in that we focused only on how lipids can influence the process of ischemic stroke through the regulation of apoptosis and did not address other cell death pathways in stroke, such as ferroptosis and pyroptosis. Furthermore, we did not undertake a comprehensive examination of the relationship between apoptosis and lipid metabolism in ischemic stroke.

## Conclusion

Various lipids can ultimately cause neuronal cell injury and apoptosis through different pathological mechanisms, such as the inflammatory response, oxidative stress, and apoptosis. These mechanisms can accelerate the pathological process of ischemic stroke and lead to a poor prognosis. Lipid metabolism disorders and lipid-induced apoptotic mechanisms in various cell types, including neurons, macrophages, and endothelial cells, are highly valuable targets for the prevention and treatment of ischemic stroke. The mechanisms linking ischemic stroke to distinct lipid metabolic pathways have been extensively investigated since lipid metabolism disorders were first considered as targets for the treatment of ischemic stroke. Numerous studies have demonstrated that dyslipidemia causes inflammation and oxidative stress and triggers the formation of “foam cells” by detaching lipid plaques and atherosclerotic lesions. This, in turn, promotes the apoptosis of stroke-associated cells and the progression of ischemic stroke. The targeted regulation of lipid metabolism disorders is expected to result in the treatment of ischemic stroke or reduce its progression by inhibiting apoptosis. Nevertheless, many questions regarding lipid metabolism remain unanswered. Lipid metabolism is a complex process regulated by multiple signaling networks. The same lipid molecule can produce different metabolites depending on the signaling pathway or condition. Therefore, understanding the relationship between multiple lipid metabolism-related signaling pathways and the acceleration of neuronal apoptosis can contribute to the unraveling of the pathophysiological mechanisms underlying ischemic stroke. Further research is needed to uncover how lipid metabolism disorders accelerate cell apoptosis and promote ischemic stroke, thereby allowing the development of new and better strategies for treating and preventing this condition.

## Data Availability

*Not applicable*.
